# 

**Published:** 1889-02-23

**Authors:** 


### CONTENTS.

**Premiums on Child-Murder** - 323

**Literature in Plague Time** - 324

**The Doctor's Pulpit.**—"All Sorts and Conditions" of Women - 325

**The Coral Islands and Fiji** - 326

**A Word for our Seamen** - 327

**Hints for the Sick-room.** By M. M. BASIL, M.A., M.B., etc.—Temperature Taking - 328

**Within the Wards.**—Cancer: A Hu dred Years Ago and To-day - 329

**Notes and News** - 330

**Everybody's Page** - 332

**Annotations.**—"Gone in for Pickles"—"The Pac that Kills" 333

**Insurance of Children** - 334

**False Impressions.** By CAROLINE FOTHERGILL, Author of "An Enthusiast," "A Voice in the Wilderness," etc. - 335

**The Student's Column.**—University of London - 337

**New Surgical Appliances** - 337

**The Editor's Letter-box** - 338

**Scraps and Gleanings. Amusements and Relaxation** 338

**EXTRA SUPPLEMENT—THE NURSING MIRROR.**

**En Passant.**—Work in the Wilds—National Pension Fund for Nurses—Nurses as Stewardesses—Miss Burford at Bolton—News from Paris—The Leeds Institution - lxxxi

**Original Articles.**—Politeness - lxxxii

**Lectures** - lxxxii

**A Unique Wedding** - lxxxiii

**The Nurses' Bookshelf** - lxxxiii

**Everybody's Opinion.**—A Letter from Jamaica—Nurses as Stewardesses.—The Combination of Private Nurses, etc. - lxxxiii

**Appointments, Vacancies, Notes and Queries** - lxxxiv

